# Do the Reclaimed Fungal Communities Succeed Toward the Original Structure in Eco-Fragile Regions of Coal Mining Disturbances? A Case Study in North China Loess—Aeolian Sand Area

**DOI:** 10.3389/fmicb.2022.770715

**Published:** 2022-04-01

**Authors:** Chuning Ji, Jiu Huang, Haochen Yu, Yu Tian, Xunzheng Rao, Xin Zhang

**Affiliations:** ^1^Engineering Research Center of Ministry of Education for Mine Ecological Restoration, China University of Mining and Technology, Xuzhou, China; ^2^School of Environment and Spatial Informatics, China University of Mining and Technology, Xuzhou, China; ^3^School of Public Policy and Management, China University of Mining and Technology, Xuzhou, China; ^4^School of Earth Sciences and Resources, China University of Geosciences, Beijing, China; ^5^Macau Environmental Research Institute, Macau University of Science and Technology, Macau, China

**Keywords:** eco-fragile region, reclamation, molecular ecological networks, fungal community, soil environmental interactions

## Abstract

Mining activity has caused serious environmental damage, particularly for soil ecosystems. How the soil fungal community evolves in mine reclamation and what are the succession patterns of molecular ecological networks still needs to be studied in depth. We used high-throughput sequencing to explore the changes in soil fungal communities, molecular ecological networks, and interactions with soil environmental factors in five different ages (the including control group) during 14 years of reclamation in eco-fragile mines. The results showed that the abundance and diversity of soil fungi after 14 years of reclamation were close to, but still lower than, those in the undisturbed control area, but the dominant phylum was *Ascomycota*. Soil nitrate-N, C/N ratio, pH, and water content significantly affected the fungal community with increasing reclamation ages. Moreover, we found that *Mortierellomycota*, despite its high relative abundance, had little significant connectivity with other species in the molecular ecological network. Fungal molecular ecological networks evolve with increasing ages of reclamation, with larger modules, more positive connections, and tighter networks, forming large modules of more than 60 nodes by age 9. The large modules were composed mainly of *Ascomycota* and *Basidiomycota*, which can form mycorrhiza with plant roots, and are not only capable of degrading pollution but are also “encouraged” by most (more than 64%) physicochemical factors in the soil environment. The results can provide a basis for scientific mine ecological restoration, especially for eco-fragile regions.

## Introduction

Microbial communities play a key role in soil function and ecosystem sustainability (Singh and Gupta, 2018), especially for the reclamation of mining areas (Jordão et al., 2021). As one of the most abundant soil microorganisms, fungi play the role of pathogens, decomposers, and symbionts in ecosystem processes and are involved in important ecological processes such as nitrogen conversion, carbon fixation, and organic matter decomposition in soil ecosystems. They are the main agents in slowing down the carbon cycle and degrading refractory organic matter (Fuhrman, 2009). Fungi are susceptible to disturbances and environmental changes and are considered to be accurate bioindicators of soil quality (Bi et al., 2019). Chen et al. (2020) showed that fungi have more biomarkers than bacterial communities and have a tighter and more complex ecological network. Soil fungal community dynamics follow a pattern that may be predictable in developing ecosystems (Cho et al., 2017).

For degraded ecosystems in general, natural restoration with longer restoration cycles can increase vegetation cover, soil nutrients, etc. However, the coal mining disturbance is so intense and persistent that the mining area can be called an extremely disturbed site, prone to serious environmental impacts (Bian et al., 2012; Michieka, 2014; Huang et al., 2018). Self-recovery is more difficult in the short term and must be guided by artificial measures in advance, using methods such as planting ecological forests to accelerate the natural succession process, which is then known as mine site ecological restoration. Ecological restoration of mining sites is often performed through revegetation, and gradual succession to a healthy and sustainable ecosystem adapted to post-mining land use (Munshower, 1994; Harris et al., 1996). However, these sites are considered to be the most unfavorable for plant growth (Lee et al., 2012), and it is difficult for soil and vegetation to improve the structure and function of mine ecosystems through natural restoration in the short term, especially that the improvement of soil properties requires a long recovery time. How to scientifically carry out the ecological restoration of mines has once become a complex problem for many mining countries.

Along with the deepening of mine ecological restoration to the micro-level, scholars have found that soil microbial communities can be used as early indicators of effective mine restoration and contribute to early ecosystem recovery (Waterhouse et al., 2014). In recent years, many scholars have also introduced fungi (e.g., AMF, arbuscular mycorrhiza fungi) into the soil restoration and revegetation process in mining areas and found that such initiatives have positive implications for early vegetation community succession (van Scholl et al., 2008; Bainard et al., 2015). In coal mining reclamation projects, fungal communities have been widely used to monitor and assess soils undergoing ecological restoration due to their sensitivity to changes in environmental conditions. Bečvář (1998) described the development of microbial diversity in six disposal sites in the Kladno coal mining area and found that species diversity increased with initial pile age and then decreased, with changes in fungal community composition and diversity. Dangi et al. (2012) described the recovery of microbial communities in reclaimed soils after coal mining disturbance over time, identifying the most important recovery phase as 0–15 years after reclamation. Not surprisingly, the recovery of soil microbial communities is crucial for successful land reclamation and ecosystem restoration (Li et al., 2015). Soil fungal diversity and structure may be a sensitive indicator of ecological stress. Therefore, if we can understand the succession pattern of soil ecosystems under reclamation, we may find the key points or species and shorten the cycle of ecological restoration.

In addition to mining areas, ecological restoration in eco-fragile regions is also a global challenge. As early as 2005, a Global Environmental Study Report, a collaboration between the United Nations and the World Bank, showed that 60% of the earth’s ecosystems are currently degraded, and more than 75% of these degraded areas belong to eco-fragile regions (UNEP, 2008). This region is often a transition zone at the junction of different ecosystems, where systems are weakly resistant to disturbance, sensitive to climate change, prone to ecological degradation, and difficult to recover themselves (Yang et al., 2018). Interestingly, eco-fragile regions and mining resources often coexist spatially; for example, China’s coal and the Middle East’s oil resources are heavily distributed in such eco-fragile regions (Charfeddine and Mrabet, 2017). In particular, China has the largest distribution of eco-fragile regions and the largest number of fragile ecological types in the world, the most typical of which is the Shendong region. The loess gullies and sandy areas here overlap with the coal production base, which accounts for more than 60% of China’s raw coal production. According to the National Energy Administration of China, as of the first half of 2018, there were 1,201 coal mines in production, with a combined annual production capacity of 2,189.49 million tons (China, 2018). In areas constrained by innate natural conditions and strongly disturbed by human activities, soil fungal communities may become essential indicators to help ecosystem recovery (Yan et al., 2018). Therefore, studying the ecological restoration of coal mining collapse sites in the eco-fragile regions of Shendong in China can provide a scientific and practical engineering basis for China and similar ecological restoration solutions of mining collapse sites in eco-fragile regions globally.

The United Nations released a large-scale global action plan in 2019 that calls for the restoration of approximately 350 million hectares of degraded ecosystems worldwide by 2030 (Waltham et al., 2020). In reality, ecological restoration is difficult, since many large-scale projects have difficulties guaranteeing vegetation restoration sustainability even after large investments. Studies have shown that soil fungi can improve the soil microhabitat and affect plant physiology through biological processes such as nitrogen fixation, phytohormone secretion, pathogen protection, and improved drought resistance of plants (Field et al., 2015; Grządziel and Gałązka, 2019; Zhang et al., 2021). It has also been shown that mycorrhizal fungi can increase plants’ phosphorus uptake, promote plant root development, increase the rate of secondary succession in plant communities, enhance vegetation restoration effects, and thus conserve biodiversity (Gillespie et al., 2015). Therefore, revealing the succession pattern of fungal communities in eco-fragile regions can be better controlled appropriately in the restoration process.

Although some patterns of fungal community succession in typical fragile regions have been demonstrated in the past (Ngugi et al., 2020), studies on fungal community succession after artificial reclamation of coal mining landslides in the region over a long period are not sufficiently advanced, and studies on the internal mechanisms of fungal communities are not sufficient. Kane et al. (2020) studied the succession of soil microorganisms from 2 to 32 years after reclamation in an open coal mine and showed that microbial biomass and diversity trend did not change significantly, but the community composition changed. Li et al. (2016) used the pyrosequencing-based approach to determine the bacterial, archaeal, and fungal phylogenetic composition in reclaimed soils following reclamation of a period between 2 and 30 years. They highlighted that fungal successional dynamics distinctly differ from bacterial and archaeal successional dynamics. However, most of these studies have analyzed succession patterns from the perspectives of soil physicochemical properties and microbial biomass, diversity, and community composition, but not the succession mechanisms within microbial communities from molecular ecology. What are the symbiotic, competitive, and antagonistic relationships among microbial populations? Which taxa play a key role? What are the connections between the intrinsic mechanisms of microbial communities and the successional patterns? These questions are still not well explained. Network analysis has proven to be a powerful method for studying complex community organization and interactions among members in different ecosystems (Luo et al., 2006; Banerjee et al., 2016; Carriger and Parker, 2021) but rarely has the successional process of artificial vegetation reclamation in mining areas been studied through the lens of molecular ecological networks (MENs). Key taxa critical for maintaining community structure and function have been neglected. In this regard, a positive correlation between taxa generally suggests the presence of cooperation, while a negative correlation might represent competition and antagonistic relationships (Chow et al., 2014). In addition, network parameters could also statistically reveal the keystone taxa that play crucial roles in maintaining microbial community structure and function (Berry and Widder, 2014; Shi et al., 2016; Banerjee et al., 2018). We conjecture that studying the interrelationships between soil environmental factors and key taxa can provide a scientific basis for enhancing reclamation techniques. Therefore, by constructing and analyzing ecological networks, we can better understand the succession pattern of soil fungi in the reclamation process, especially the association between different taxa.

Although high-throughput sequencing techniques and network analysis methods provide efficient and convenient means for microbial analysis, there are difficulties with our time series analysis (i.e., no equal time interval due to factors such as planning of mined coal and different techniques for reclamation). We still strive to ensure that other confounding factors such as topography and slope are not brought into the sampling process as much as possible, while our research focuses on the patterns of succession rather than on precise time points (Hatje et al., 2016). In this study, using plantation soils of different reclamation ages as the research object, we hypothesized that (1) the fungal diversity in soils after reclamation increases with the increase in reclamation years, (2) the biomarkers in soil fungal communities are different at different reclamation stages, (3) there is a succession pattern in fungal MENs, (4) the succession of soil fungal communities is related to the soil physicochemical properties, and (5) the soil fungal communities succeeded in the original structure in diversity, but not in the original structure in the MENs.

## Materials and Methods

### Site Description and Sample Collection

This study was conducted in the central Shendong coal mining, inner Mongolia, China (39°33′45″–39°11′34″ N 109°49′51″–110°20′12″ E). The study site is located in the southeast of Ordos Plateau, the northern edge of Loess Plateau in Northern Shanxi, and the southeast edge of Maowusu Desert, about 900 km^2^. It is located in the transition zone between dry grassland and forest-steppe with the typical arid and semi-arid plateau continental climate. The annual average temperature is 6.2°C, the extreme minimum temperature is 1.4°C, and the extreme maximum temperature is 36.6°C. The annual precipitation is about 300–400 mm, mainly concentrated in summer; evaporation is more than four times the rainfall.^1^ The landform is high in the northwest and low in the southeast, and we measured an average elevation of about 1,200 m using a GPS receiver (CHC I50). In the coal mine sinkhole area of Mauwusu Sandy, *Hippophae rhamnoides* can adapt to both adverse conditions of harsh natural environment and sinkhole, as Wu et al. (2021) demonstrated that the ecological restoration effect of long-term reclaimed *H. rhamnoides* forest is better. The diversity and richness of soil fauna are increasing. After growing into a dense forest at the bottom of the ditch, *H. rhamnoides* is extremely resistant to rainwater erosion and is not afraid of wind and sand blowing and burying and has a robust root system that can stop storm erosion and flooding, effectively intercept sediment, and also improve the erosion reference surface on both sides of the ditch (Bolibok et al., 2009). Therefore, in this study, the soil samples were selected nearby the root of *H. rhamnoides*.

**FIGURE 1:**
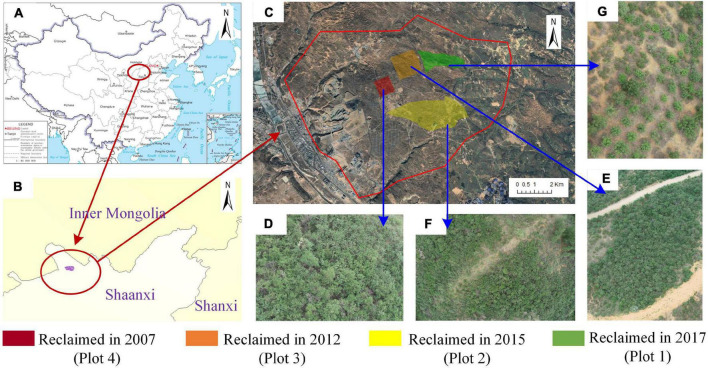
**(A,B)** The geographic locations of the study area, **(C)** the area sampled, and **(D–G)** photographs of *Hippophae rhamnoides* that were reclaimed in 2007, 2012, 2015, and 2017, respectively.

Soil samples were collected from five plots: one reference plot with native *H. rhamnoides* (CK); the other four plots with different times since reclamation, that is, 4 (Plot 1), 6 (Plot 2), 9 (Plot 3), and 14 (Plot 4) years ago, which were artificially planted with *H. rhamnoides* (Figure 1). As shown in Figure 1, after 14 years of reclamation, *H. rhamnoides* grew more densely and taller (Plot 4). On the contrary, the *H. rhamnoides* was smaller and sparser, where only 4 years of reclamation had passed (Plot 1). The growth of *H. rhamnoides* after 6 and 9 years after reclamation was in between (Plots 2 and 3). These five soil samples were collected at four different years to study the succession pattern of soil fungal communities in the coal mining subsidence reclamation area over 14 years (Chao et al., 2016). To avoid, as far as possible, interference from potential influences such as topography, each soil sample was sampled in a neutral zone of collapse (slope of almost 0°). All sampling instruments were sterilized and disinfected beforehand. In each sample plot, surface impurities were removed, and the soil around five *H. rhamnoides* plants with a depth of about 5 cm was randomly dug and mixed to form one sample. Twelve samples were collected from each of the five sample plots, totaling 60 samples, and transported to the laboratory on ice. The soil water content (SWC) was determined with a portable hygrometer. On the day of collection, visible roots and other material were sieved through a 2-mm sieve (Vishwakarma et al., 2020), transported to the laboratory in cold storage, and divided into two subsamples for microbiological and physicochemical analysis at 4°C. The soil subsamples were placed in extraction buffer on the same day for DNA extraction and stored at −80°C if required during the process.

### Soil Physicochemical Analyses

Soil pH and electrical conductivity (EC) were determined by the potentiometric method (water/soil = 1: 5) with DDS-307A (INESA, Shanghai, China). The mechanical composition of soil and the proportion of clay (PC) were determined by a 311XP particle size analyzer (WINNER, Zhejiang, China). Soil total nitrogen (TN) contents were determined with the Nky-6120 Automatic Kjeldahl nitrogen analyzer (YIHON, Shanghai, China). Nitrate-nitrogen (NN) was determined by phenol disulfonic acid colorimetry, and ammonium nitrogen (AN) was determined by starch blue colorimetry (LU/T 1228-2015). The potassium dichromate oxidation external heating method used the soil organic carbon content (SOC) and C/N ratio (LY/T 1237-1999). The contents of total phosphorus (TP) and kalium (TK) in soil were determined by the alkali fusion method (LY/T 1232-2015 and LY/T 1234-2015).

### DNA Extraction and Sequencing

Total DNA was extracted from soil samples collected from each reclaimed plot with the Soil DNA Kit. The internal transcribed space (ITS) rDNA amplicon sequencing that we used includes the library construction using specific primers (PE read 1 sequencing primer, 5′ ACACTCTTTCCCTACACGACGCTCTTCCGATCT, and PE read 2 sequencing primer, 5′ CGGTCTCGGCATTCC TGCTGAACCGCTCTTCCGATCT) to amplify the variable region of eukaryotic ITS rDNA and data analysis to identify the composition and abundance of eukaryotic microorganisms in the soil samples. The adapters input into Cutadapt is AGATCGGAAGAGC, and the adapters information sequenced by Miseq PE300 is P7 adapter (read), AGATCGGAAGAGCACACGTCTGAACTCCAGTCAC, and P5 adapter (read2), AGATCGGAAGAGCGTCGTGTAGGGAAA GAGTGT.

The original image data were analyzed using Bcl2fastq (v2.17.1.14) for base calling and preliminary quality analysis. During sequencing, Illumina’s built-in software determines whether to keep or discard each sequenced segment based on the quality of the first 25 bases of the read. The quality of the base determines whether the read is retained or discarded. Moreover, the two sequences of each read pair were merged according to overlapping sequences. The read merge is deemed to be successful only if the overlapping sequence is at least 20 bp long. After merging, undetermined bases (N) were removed from the resulting sequence. Then, primer and adapter sequences were removed, and the 5′ and 3′ bases with Q ITSscore lower than 20. The resulting sequences with a length > 200 bp would pass this processing step. We finally got the effective data after the sequences were aligned to a database to identify and remove the chimera sequence.

Unique sequences are extracted from the optimized sequences with the read count information and removed the unique sequence with one read count. Operational taxonomic unit (OTU) clustering of unique sequences (read count > 1) was performed with a similarity of 97%, and chimeric sequences were further removed to obtain the representative OTU sequences. All optimized sequences are compared with OTU representative sequences, and sequences of > 97% similarity to a specific OTU representative sequence are considered to be of the same OTU and then summarized the OTU abundance.

### Network Construction and Analysis

In this study, a random matrix theory (RMT)-based approach was used to construct the network. The RMT theory assumes two extreme distributions of matrix eigenvalues of the distribution of the nearest-neighbor space: the Gaussian orthogonal ensemble (GOE) distribution and the Poisson distribution. The former corresponds to complex stochastic systems applicable to a particular system. The eigenvalues of their adjacency matrices are calculated by varying the threshold values and obtaining the adjacency matrices corresponding to different thresholds. This threshold is considered reasonable if the eigenvalue distribution approximates the Poisson distribution and continues to be adjusted if it approximates the GOE distribution. The final threshold is considered a reasonable threshold for building the network. This way of constructing the network can automatically obtain the threshold value and eliminate the noise interference in the data (Zhou et al., 2010, 2011; Deng et al., 2012). The networks were separately constructed based on ITS gene sequence data for four plots in a reclaimed mining area. The framework for network construction using the Molecular Ecological Network Analyses Pipeline^2^ can be divided into four key steps: (1) metagenomic sequence collection, (2) data standardization, (3) pair-wise similarity estimation, and (4) adjacent matrix determination. Compared to other network construction methods, the distinctive feature of this approach is that the network is automatically defined and highly robust to noise (Deng et al., 2012). The network module comprises highly interconnected nodes (OTUs in this study) with few connections outside the group. Modularity describes the extent to which nodes attain more links within their modules than expected for random linkages. Modules were detected by the greedy modularity optimization method. Network topology analysis is performed, including network topology characterization, module detection, module-based feature gene analysis, and module role identification. We calculated a series of basic metrics to describe the topology of the network, including network nodes, edges, average clustering coefficient (avgCC), average connectivity (avgK), average geodesic distance (GD), and model number (M). Then, the gene/OTU significances (GS) with environmental traits were calculated. The Mantel test to correlate GS and network connectivity was run after the soil variable matrix was uploaded and Euclidean distance was selected (Horner-Devine et al., 2004). Finally, Maslov–Sneppen method (Maslov and Sneppen, 2002) was used to reconnect nodes at different locations in the original network without changing the number of nodes and connections in the original network. The difference in fungal network structure between different regions was analyzed by *t*-test. The above analyses were carried out using the Molecular Ecological Network Analyses pipeline,^2^ visualizing MENs with Gephi 0.9.2 (Goslee and Urban, 2007).

### Statistical Analysis

ANOVA analysis and Tukey were used to test the diversity index of soil physical and chemical parameters and the fungal community whether there was a significant difference (*p* < 0.05). All statistical analyses were performed on natural logarithm (log10)-transformed data to normalize abnormally distributed data. OTU analysis and species annotation were analyzed by Qiime (1.9.1), and Vsearch (1.9.6), the Shannon index, Simpson index, and dilution curves were analyzed using Qiime (1.9.1) as well. We conducted a characterization using redundancy analysis (RDA) to explore the correlation and driving mechanisms between soil fungal community structure and environmental factors after the reclamation of coal mining subsidence areas. RDA is a principal component analysis of environmental factors, which can reflect the microbial community and environmental factors in the same two-dimensional sequence diagram, from which the relationship between microbial distribution and environmental factors can be seen directly (Huhe et al., 2017). All statistical analysis and mapping were performed using R (V3.6) (R Core Team, 2011) and Origin.

## Results

### Soil Physicochemical Characteristics

Land reclamation of coal mining subsidence sites significantly affected soil physicochemical properties (*p* < 0.05) (Table 1). Soil pH tended to decrease with increasing reclamation ages but was higher than CK, which was almost neutral, with significant differences between CK and Plot 4 and Plot 3, Plot 2, and Plot 1 (*p* < 0.001). SWC was highest in Plots 4 and 3, close to CK, and significantly higher than Plots 2 and 1 (*p* < 0.001). EC was highest in Plot 4 and was significantly different from CK, Plot 3, Plot 2, and Plot 1, respectively. The proportion of clay (PC) increased with the number of ages of reclamation, with no significant difference between CK and Plot 2, and it was significantly higher in both Plots 4 and 3 than in CK (*p* < 0.001). SOC was highest in Plot 3, with no significant difference between Plot 4 and CK and significantly lower in Plot 2 and Plot 1 than in other soils. TN and NN were highest in Plot 4 and were significantly different from all other samples (*p* < 0.001). The soil C/N ratio had the highest value in Plot 1 and the lowest in Plot 4 and was quite different (*p* < 0.001). TK and TP in soil were not significantly different in any five groups.

**TABLE 1:** Physicochemical properties of the soil in different reclamation ages.

Sample name	Physicochemical parameters					
	pH	SWC (%)	EC (μS/cm)	PC (%)	SOC (g/kg)	C/N
CK	7.25 ± 0.18b	8.96 ± 1.21b	32.83 ± 5.00c	23.56 ± 2.56b	4.08 ± 0.89b	7.85 ± 4.33c
Plot 4	7.86 ± 0.16b	13.54 ± 0.36a	72.01 ± 13.22a	26.41 ± 3.18a	6.33 ± 0.56a	5.28 ± 2.16d
Plot 3	7.83 ± 0.12b	11.58 ± 0.56a	59.15 ± 13.14b	24.84 ± 4.89a	7.10 ± 1.23a	11.09 ± 1.58a
Plot 2	8.18 ± 0.39a	10.34 ± 0.39b	41.30 ± 14.91b	22.62 ± 1.89b	5.94 ± 0.06a	9.90 ± 2.56b
Plot 1	8.24 ± 0.88a	6.60 ± 0.02c	28.32 ± 3.3c	18.37 ± 4.65c	2.09 ± 0.21c	13.06 ± 2.47a

**Sample name**	**Physicochemical parameters**					
	**TN (g/kg)**	**AN (mg/kg)**	**NN (mg/kg)**	**TP (g/kg)**	**TK (g/kg)**	

CK	0.52 ± 0.03b	4.51 ± 1.10b	13.83 ± 2.5c	0.48 ± 0.01a	16.71 ± 2.68a	
Plot 4	1.20 ± 0.04a	4.37 ± 0.89b	29.62 ± 4.52a	0.56 ± 0.04a	16.12 ± 6.10a	
Plot 3	0.64 ± 0.06b	6.91 ± 0.43a	19.61 ± 5.50b	0.60 ± 0.21a	16.01 ± 1.54a	
Plot 2	0.60 ± 0.01b	5.07 ± 0.14a	14.51 ± 2.59c	0.46 ± 0.45a	16.20 ± 2.89a	
Plot 1	0.16 ± 0.01c	2.73 ± 0.02c	7.90 ± 0.43d	0.35 ± 0.64a	17.01 ± 3.56a	

*CK represents soil in a reference plot with native Hippophae rhamnoides vegetation on the mine site, and Plot 4, Plot 3, Plot 2, and Plot 1 represent soils with Hippophae rhamnoides vegetation reclaimed on the mine site in 2007, 2012, 2015, and 2017, respectively. The values were presented as mean ± standard error of the mean (S.E.M). Different lowercase letters indicate significant differences in different samples (p < 0.05).*

### Fungal Community Composition and Diversity

**FIGURE 2:**
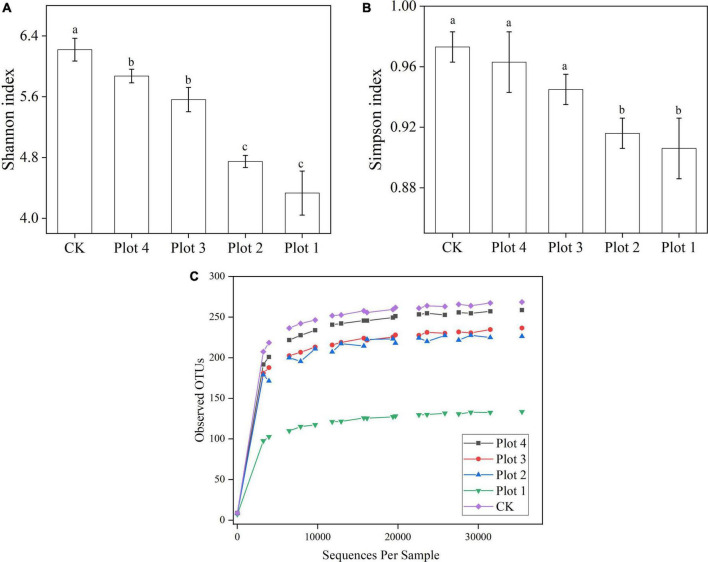
**(A)** Shannon index, **(B)** Simpson index, and **(C)** observed OTUs rarefaction curves of soil fungi in sample plots with different reclamation ages. CK represents soil in a reference plot with native *Hippophae rhamnoides* vegetation on the mine site, and Plot 4, Plot 3, Plot 2, and Plot 1 represent soils with *Hippophae rhamnoides* vegetation reclaimed on the mine site in 2007, 2012, 2015, and 2017, respectively. Values in the table are means ± standard deviation. Different lowercase letters in the same column represent a significant difference at 0.05 level.

A random sampling of the sequences was used to construct a Rarefaction curve with the number of sequences drawn vs. the number of OTUs they could represent (Figure 2C). At fungal sequencing sequences > 25,000, the curves gradually leveled off for each sample, representing sufficient sequencing depth and a reasonable amount of data to reflect each sample’s soil fungal species and structure. The species richness of CK was the highest, and the species richness of the other samples gradually increased with the increase in reclamation ages, in the order of Plot 4 > Plot 3 > Plot 2 > Plot 1. The species richness of Plot 1 was lower than that of the other four groups, which might be due to the low reclamation ages, soil restoration, and vegetation growth greatly influenced by human factors at the early stage of reclamation and entered the natural recovery stage later. In this study, the Shannon and Simpson indexes were used to characterize the fungal community diversity. As seen in Figures 2A,B, for Shannon index, CK > Plot 4 > Plot 3 > Plot 2 > Plot 1, and for Simpson index, CK < Plot 4 < Plot 3 < Plot 2 < Plot 1. CK and Plots 1 and 2 were significantly different.

**FIGURE 3:**
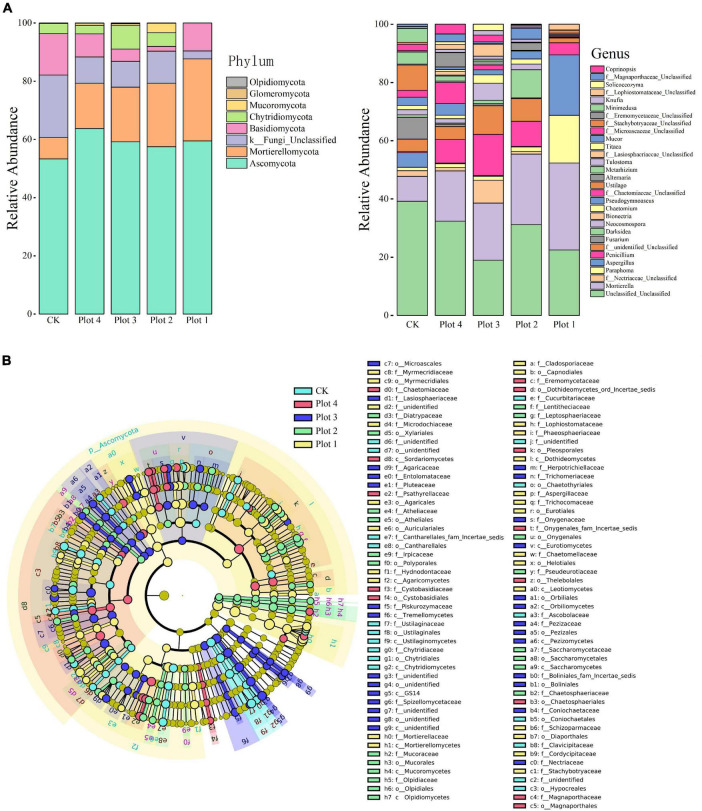
**(A)** Structural composition of soil fungi at phylum and genus level. **(B)** LEfSe of the fungal communities with an LDA score higher than 4.0. In cladogram, circles radiating from inside out represent fungal taxa from phylum to genus, the diameter of which is proportional to the relative abundance of each taxon. Taxa with significant differences were marked with the same color as the highest-ranked sampling point of that taxa, and the branching regions were shaded. A taxon without a significant difference is marked in yellow. CK represents soil in a reference plot with native *Hippophae rhamnoides* vegetation on the mine site, and Plot 4, Plot 3, Plot 2, and Plot 1 represent soils with *Hippophae rhamnoides* vegetation reclaimed on the mine site in 2007, 2012, 2015, and 2017, respectively.

At the phylum level (Figure 3A), the structural composition of the soil fungal community was similar across the samples, but the relative abundance of each group varied significantly. *Ascomycota* was the most abundant dominant phylum, followed by *Mortierellomycota* and *Basidiomycota*. The relative abundance of *Ascomycota* was 53% in the native *H. rhamnoides* soils and ranged from 57 to 64% in the reclaimed soils, similar to CK. *Mortierellomycota* had the lowest relative abundance of 7% in CK. In contrast, its relative abundance was higher than CK in all reclaimed soils, and the relative abundance became lower as the number of years of reclamation increased. In contrast, the relative abundance of *Basidiomycota* was highest in CK, followed by Plot 2, Plot 3, Plot 4, and Plot 1, while *Chytridiomycota* was highest in Plot 3, followed by Plot 2, Plot 4, and CK, and almost absent in Plot 1. *Mortierella*, *Paraphoma*, and *Aspergillus* were the three dominant genera with the highest abundance. Meanwhile, in Plot 1, the species richness was significantly lower than the other groups, with the dominant species mainly being *Mortierella*, *Paraphoma*, *Aspergillus*, and *Penicillium*, occupying more than 93% of the abundance, while the rest of the genera were less diverse and less abundant. This is consistent with the results of the above dilution curves, which show that all genera in CK are more diverse and even in relative abundance. It can be seen that the structure and relative abundance of fungal communities at the phylum level differed between the reclaimed areas and those unaffected by mining at different ages.

Linear discriminant analysis (LDA) effect size analysis (LEfSe, LDA effect size) was used to identify the different biomarkers with LDA scores > 4.0 (Figure 3B). These biomarkers showed significant variation in the relative abundance of core microorganisms and changed significantly with reclamation and age. A total of 102 significantly different microbial taxonomic units were obtained, i.e., five groups of key biomarkers of fungal communities at the microbiological taxonomic level were obtained. The results showed that CK, Plot 4, Plot 3, Plot 2, and Plot 1 had 18, 13, 27, 20, and 24 key biomarkers, including 33 orders, 13 class, and 56 families, respectively. In addition, the microbial markers at all taxonomic levels were from *Ascomycota*, *Mucoromycota*, *Mortierellomycota*, and *Olpidiomycota*. In the five sample plots, Plots 4 and 3 were similar, with only *Ascomycota* as a microbial marker, indicating that the distribution of microbial species in these two groups was relatively homogeneous, with no obvious markers, while *Mortierellomycota* was a marker only in Plot 1, and this phylum will no longer be iconic with increasing reclamation ages. In addition, *Basidiomycota* and *Chytridiomycota* were not represented and can be considered to have insignificant intergroup variability. The most significant taxa in the CK samples were *Ustilaginomycetes* (phylum to species); the most significant taxa in the Plot 4 samples were *o_leosporales*, *f_Chaetomiaceae*, and *f_Psathyrellaceae*; the most significant taxa in the Plot 3 sample were *GS14* (phylum to species) and *f_Nectriaceae*; the most significant taxa in the Plot 2 sample were *Darksidea* (genus to species) and *f_Lentitheciaceae*; and the most significant taxa in the Plot 1 sample were *Mortierellomycota* (phylum to species), *Aspergillaceae* (family to species), *g_Paraphoma*, and *o_Eurotiales* (LDA score > 5.0), indicating that the diverse composition of the microbiota varied with reclamation and the number of ages of reclamation.

### Correlation of Soil Environmental Factors With Fungal Communities

**FIGURE 4:**
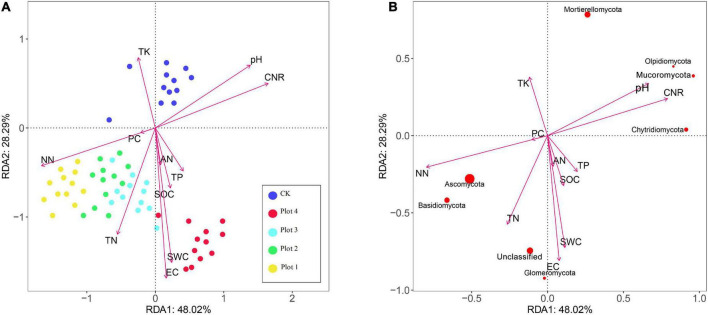
Redundancy analysis (RDA) of the relationships between soil physicochemical properties and fungal communities (*p* < 0.01). **(A)** Shows the relationship between soil physicochemical factors and soil sample distribution, and **(B)** shows the relationship between the major fungal phyla and soil physicochemical factors. The size of the point represents the abundance of the species. Percent variability explained by each principal component is shown in parentheses after each axis legend. CK represents soil in a reference plot with native *Hippophae rhamnoides* vegetation on the mine site, and Plot 4, Plot 3, Plot 2, and Plot 1 represent soils with *Hippophae rhamnoides* vegetation reclaimed on the mine site in 2007, 2012, 2015, and 2017, respectively.

Detrended correspondence analysis (DCA) of the soil fungal taxa yielded a maximum value of 0.199 for the axis gradient, indicating that the linear model (RDA) was chosen for better results. RDA can be a favorable way to assess the correlation between microorganisms and environmental variables, using Canoco 5.0; the results of the RDA analysis are shown in Figure 4. Of the RDA1 and RDA2 axes, 48.02 and 28.29% were, respectively, explained by the soil fungal community level and environmental factors in the control area and at four different reclamation years. The soil environmental factors that correlated well with the distribution and composition of the fungal community at different reclamation years were NN, EC, SWC, C/N ratio, TN, and pH. The order of magnitude of the effect of NN on the soil fungal community was Plot 1 > Plot 2 > Plot 3 > Plot 4≈CK. The order of magnitude of the effect of SWC and EC on the fungal community was Plot 4 > Plot 3≈Plot 2 > Plot 1 > CK. The effect of TN on the fungal communities of the four reclaimed soils was similar in magnitude and more significant than the effect on CK. TK had the most significant effect on CK and less on the reclaimed soils.

The cosine between the straight line from each species to the origin and the environmental factors represents the correlation. As seen in Figure 4B, soil NN concentrations were significantly positively correlated with the relative levels of *Ascomycota* and *Basidiomycota* and negatively correlated with *Mucoromycota*, *Olpidiomycota*, and *Chytridiomycota*. SWC and EC were significantly positively correlated with *Glomeromycota* and *unclassified* and negatively correlated with *Mortierellomycota*. Soil pH, C/R ratio, and NN concentration were significantly and negatively correlated with *Ascomycota*, *Basidiomycota*, and *Mucoromycota*, *Olpidiomycota*.

### Molecular Ecological Networks and Interactions of Fungi Under Different Reclamation Ages

**FIGURE 5:**
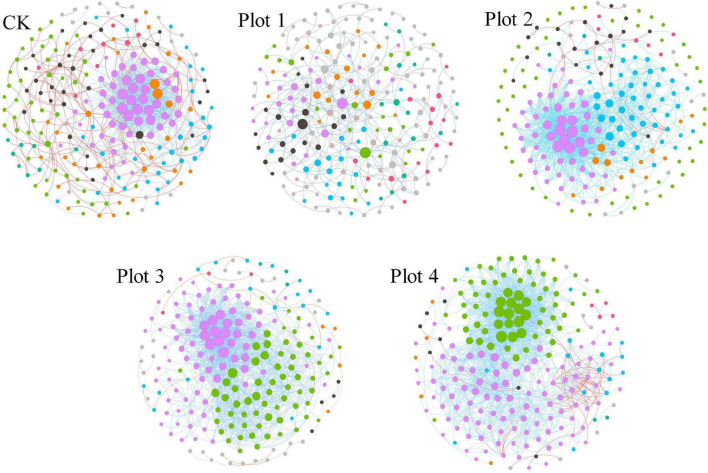
Soil fungal MENs and interaction. Nodes were colored by different modules; the size represents the node degree (Connectivity). The connection line represents the interaction between the target level on phylum, with blue representing positive and red representing negative.

Identifying modules in complex networks can reveal the organization of these networks and how they further become tightly linked communities, as they form independent or linked modules in the community according to the interactions between microbial species. MENs between fungal species were constructed by screening and retaining OTUs that co-occurred in 70% of the samples (Figure 5). MENs were built at the same similarity threshold of Pearson (0.890) for different soil reclamation years, allowing direct comparison of the topology coefficients of the different networks (Table 2). Each MENs in different reclamation ages is divided into discrete modules containing closely related prokaryotic taxa, where coexisting taxa are considered to share environmental preferences or interact in a mutually reinforcing manner. A node represents an OUT, and each edge represents the correlation between the two nodes connected by it. The overall topological index shows that all network connectivity distribution curves fit well with the power-law model (*R*^2^ between 0.731 and 0.863), indicating the scale-free property of the network. This implies that fewer fungi in the MENs were more connected to other fungi, while most were less connected. These network topology indices were significantly different from random networks with the same number of nodes and edges (*p* < 0.01, Table 2), indicating that the network structure was not constructed randomly and without order and that their interconnections were significantly deterministic. The average path length (GD, a value measuring the efficiency of information or quality transfer on the network) of the molecular ecological network for the five sample sites ranged from 3.156 to 4.979, which is comparable to the GD of networks showing small-world behavior summarized by Brown et al. (2004) and Deng et al. (2012), indicating that the network constructed here is small-world in nature.

**TABLE 2:** Topological properties of the empirical ecological networks.

Plot	Threshold for Pearson	No. of nodes	Total links	*R*^2^ of power law	Average connectivity (avgK)	Average geodesic distance (GD)	Average clustering coefficient (avgCC)	Modularity (M)/no module
CK	0.890	232	818	0.810	7.052	4.852	0.130	0.386/15
Plot 4	0.890	214	1,609	0.826	15.037	3.156	0.302	0.399/11
Plot 3	0.890	209	1,368	0.731	13.091	3.663	0.350	0.351/11
Plot 2	0.890	158	975	0.777	12.342	3.464	0.377	0.312/12
Plot 1	0.890	224	293	0.863	2.616	4.979	0.044	0.728/26

The degree of connectivity of the soil fungal interactions network varied between reclamation and control areas unaffected by mining subsidence (Figure 5). Various network topology indices consistently showed that the interconnections of soil fungi differed considerably between sites (Table 2). More nodes and edges mean more extensive networks between fungi, and their connections are likely to be more complex. Therefore, there are fewer total links in Plot 1, resulting in separated networks from each other (Figure 5). Average connectivity (avgK), average clustering coefficient (avgCC), modularity (M), and average geodesic distance (GD) consistently reflect a trend of increasing network complexity and connectivity with increasing reclamation age for Plots 4, 3, and 2, reflecting that the fungal MENs are becoming progressively more compact. The number of nodes in the Plot 1 ecological network increased, but the number of connecting lines decreased, indicating that the diversity of the fungal MENs increased. However, the interconnectedness decreased, which may be related to the formation of soil agglomerates or the reconfiguration of microbial functional groups after reclamation.

In Figure 5, the blue lines represent positive interactions between two nodal OTUs, and the red lines represent negative interactions. In Plots 4, 3, and 2, several major modules cooperate much more than compete. The OUT with a high node degree in CK forms the two most dominant modules, and the OTUs between modules cooperate or have the same ecological niche. However, the other non-major modules and OUTs had the most competitive relationships among the five networks. The reclaimed network OUTs and modules generally cooperate with each other more than they compete. This suggests that soil fungi may prefer to cooperate to adapt to mutated environments or have similar ecological niches during land reclamation.

**FIGURE 6:**
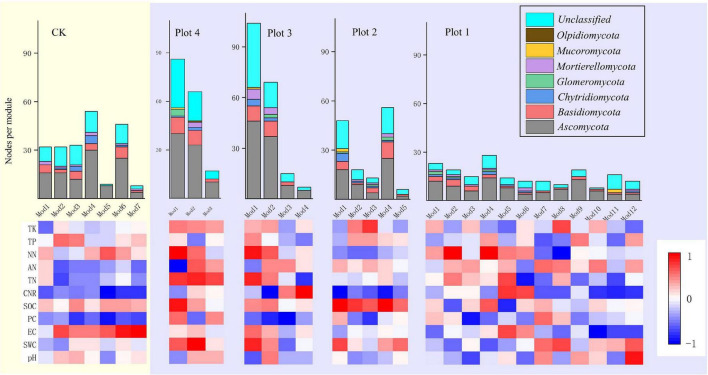
Heat map of correlation between soil parameters and eigenvalues of soil fungal molecular ecological network modules (number of nodes > 5) with node taxonomy for different reclamation ages. Red represents positive correlation, blue represents negative correlation, and the histogram shows the number of nodes in each module and the phylum they belong to.

Module-EigenGene analysis using greedy modularity optimization revealed the relationship between all modules with more than five nodes and each soil environmental variable (Figure 6). Mod 1 and mod 4 in Plot 2 were significantly negatively correlated with the C/N ratio and significantly positively correlated with SOC in the soil. Mod 1 and Mod 2 in Plot 3 had more than 60 nodes, and all were positively correlated with NN. Mod 1 in Plot 4 was significantly positively correlated with NN, significantly negatively correlated with pH, and Mod 2 was significantly negatively correlated with AN (*p* < 0.01). Each module in the soil fungal ecological network of the five sample plots contained *Ascomycota*, a dominant phylum. In Plot 1, all 10 modules were positively correlated with AN, except Mod 4 and Mod 11, and all seven modules in CK were negatively correlated with the C/N ratio.

## Discussion

This paper used the powerful and efficient platform provided by Illumina MiSeq high-throughput sequencing technology to analyze the microbial community structure (Barberan et al., 2014), composition, and molecular ecological network to answer the question posed by the title: the fungal communities in different reclamation ages of coal mining subsidence in ecologically fragile areas did not evolve along the original direction but a secondary succession. The influence of SWC, pH, SOC, and C/N ratio on fungal communities increased with reclamation time, while the influence of NN was significant but decreased with the reclamation time. The stability and structure of the MENs and the biomarkers of the different stages evolved along a new direction. This conclusion provides a new reference for refining the rapid and effective application of microbial remediation technology to land degradation and ecological restoration in mining areas.

### Successional Directions of Fungal Community Diversity and Composition Structure

It has been suggested that the overall composition of microbial communities may vary considerably across habitats, but the dominant groups are primarily similar (Zak et al., 2003). This study also found that the dominant soil fungal phyla under four different reclamation years were *Ascomycota* and *Mortierellomycota*, with the relative composition of the two groups of fungi exceeding 60% as the dominant phyla. The soils surrounding the mines contain a large number of coal substrates that consist of a complex mixture of aromatic hydrocarbons and aliphatic and lignin-derived macromolecules (Po et al., 2011). It was previously observed that these dominant gates might promote methanogenic bacteria metabolism for methanogenesis and coal degradation (Wagg et al., 2019). In previous studies, members of *Ascomycota* have been observed to exhibit a high degree of effectiveness in degrading polycyclic aromatic hydrocarbons (PAHs) (Guo et al., 2018). The presence of these fungi is an interesting result, indicating the potential for biodegradation of mine soil coal under the right conditions (Chen et al., 2020). *Ascomycota* abundance was all-around 60% with little difference, but *Mortierellomycota* abundance varied across the five sample groups: in the reclaimed mine sample sites, abundance increased with decreasing reclamation years (16–28%) and was lowest in the unreclaimed control sample sites (around 7%). *Mortierellomycota* was the marker at the 2017-year reclaimed soil clade level in the lefse analysis. At the generic level, all five sample plots contained many unidentified fungal genera, with *Mortierella* being the most abundant of the identified fungal genera and decreasing in abundance in the reclaimed plots from 2017 to 2007. *Mortierella* strains are the most abundant filamentous fungi in the soils around the world (Domsch et al., 1982; Smit et al., 1999; Stefania et al., 2012; Wu et al., 2013; Raj et al., 2015; Qiao et al., 2018; Grządziel and Gałązka, 2019), and they are the most common soil-adapted fungi that can survive and reproduce at low temperatures or in nutrient-poor environments. Filamentous mycelium stabilizes the soil structure, penetrating soil pores, and solid substrates such as rocks and minerals into cavities, crevices, and fissures, which undergo physical changes related to structure and size. For the mycelium to occupy large areas, they secrete acidic compounds used to bore micro-channels in a stable environment (Daghino et al., 2008; van Scholl et al., 2008). In ecologically fragile regions, poor soil structure and heterogeneity at the beginning of coal mining reclamation may have caused *Mortierella* to be more widely distributed in soils with a short reclamation age.

In general, the soil moisture content is one of the crucial factors affecting microbial community richness (Ridolfi et al., 2008), especially in eco-fragile regions. The SWC of the sample plots reclaimed in 2015 and 2017 was significantly lower than that of the sample plots reclaimed in 2007 and 2012, and the control sample plot (*p* < 0.01), resulting in significantly lower fungal community richness and diversity. In this study, the main phyla of fungi in the semi-arid mine reclamation soil were *Ascomycota*, *Mortierellomycota*, and *Basidiomycota*, which are similar to the main phyla of fungi in terraced soils of the Loess Plateau (Buckley and Schmidt, 2001), acidic soils of tea plantations in Yunnan (Sun et al., 2016), and oil sludge-contaminated soils in the Yellow River Delta (Wang et al., 2018). These results indicated that these fungi existed widely in soil and were not unique to semi-arid eco-fragile areas. The relative abundance of *Basidiomycota* and *unidentified* phyla is higher in unreclaimed sites than in other reclaimed soils, and some studies have estimated fungal species worldwide, with < 0% being formally described (Hawksworth and Rossman, 1997), making an in-depth study of unidentified fungal species important. The greater number of fungal species compared to unreclaimed sites resulted in more intense competition for energy and nutrients between species, and the reduction in the relative abundance of the major clades of fungi in reclaimed soils between 2017 and 2007 (*Ascomycota* + *Mortierellomycota*) is further evidence that revegetation enriched the community structure of soil fungi. Despite the impact of vegetation restoration on soil fungal community structure, the soil fungal community structure remained somewhat stable between the four sample sites, suggesting that ecological restoration of reconstructed soil fungal communities in eco-fragile mining regions is a long process.

### Interaction Mechanism of Soil Fungal Communities and Environmental Factors

Before exploring the relationship between fungal communities and the enhancement of soil properties, we found significant differences in soil physicochemical properties under different reclamation ages. The spot reclaimed in 2007 outperformed the other 3 years in most indicators and the control sample, which was unaffected by mining (Table 1). Similar to the results of previous studies, revegetation plays an important role in promoting and restoring the ecological environment in fragile regions (Wang et al., 2016; Yang et al., 2016). Under the influence of several biochemical processes, shrub soils in arid and semi-arid areas usually have a higher soil nutrient content, a phenomenon known as the “Waldo effect” (Ridolfi et al., 2008). The effectiveness of soil nutrients is closely related to soil microorganisms, whose behavior influences the mineralization of soil nutrients, and revegetation can change the habitat of microorganisms (Wang et al., 2021a). Thus, the effectiveness of ecological resources is urgently needed in ecologically fragile areas.

In this study, the results of the RDA of the fungal community structure and soil physicochemical factors in soil samples from different reclamation ages and control areas (Figure 4A) showed that the soil fungal community composition was similar for the reclamation in 2012, 2015, and 2017, but there were significant differences between the control and the reclaimed soil fungal communities. The main factors affecting the soil fungal community in the different reclamation years were NN, EC, SWC, C/N ratio, TN, and pH (*p* < 0.05). This was also reflected in the results of the RDA of dominant fungal phyla and soil physicochemical factors (Figure 4B). Soil NN was most correlated with fungal communities and significantly correlated with four phyla: the dominant phyla *Ascomycota* and *Basidiomycota* were closely related phylogenetically, and they both formed mycorrhiza in symbiosis with plants, which facilitates the growth and development of vegetation. Li et al. (2013) found that key competition for nutrients between vegetation and microbes took place during the initial reclamation period, with soil nutrients representing a limitation of the microbial community during reclamation succession. N-cycling microbes can compete with plants for available soil nutrients, exhibiting negative interactions with the plant community (Hodge and Fitter, 2013; Blagodatskaya et al., 2014; Wang et al., 2021b). Some fungi can alter plant community composition and affect inter-root and mycorrhizal inter-root microbial community composition (Rillig, 2004). For example, AMF induce shifts of N-cycling processes, such as nitrification, by altering the physiology of the host plant and changing the inter-root sediment (Veresoglou et al., 2019). Zhang et al. (2019) showed that earthworms and AMF fungi’s combined effect increased soil ${\text{NO}}_{3}^{-}$-N and ${\text{NH}}_{4}^{+}$-N. Their result suggested that earthworms and AMF could stimulate prokaryotic growth and bacterial nitrification by improving soil macroaggregate formation in saline soil. The above studies support our suggestion that the role of soil properties on fungal communities may depend mainly on the level of nitrogen nutrients in the reclaimed soil.

The fungal phyla that are significantly positively correlated with pH and C/N ratios are *Mucoromycota* and *Olpidiomycota*. *Mucoromycota* with their three subphyla (*Glomeromycotina*, *Mortierellomycotina*, and *Mucoromycotina*) group species of mycorrhizal fungi, root endophytes, plant pathogens, and many decomposers of plant material. The group of Katie Field has shown (Field et al., 2015) that *Mucoromycotina* harbors a great deal of undescribed diversity and is a relevant source of fungi that interact with plants. The capacity to colonize plant tissues and produce arbuscules is present in both *Mucoromycotina* and *Glomeromycotina*, while the ectomycorrhizal traits are shared with many other fungal taxa from *Basidio-* and *Ascomycota* (Tedersoo and Smith, 2013). In addition to *Unclassified*, *Glomeromycota* can constitute the arbuscular mycorrhizas (AM) of terrestrial plants and is therefore called “AM fungi,” which are positively correlated with soil EC and SWC. This symbiotic structure assists plants in the uptake of inorganic salts from the soil, particularly phosphorus (Brundrett, 2002). As the physical properties of the soil improve over the years of reclamation, the clay content of the soil increases, water retention increases, and the water content of the soil increases (Table 1). *Glomeromycota* may gradually become the dominant fungus.

### Discussing the Direction and Mechanism of the Succession From the Perspective of Molecular Ecological Networks

The internal interrelationships between microorganisms cannot be characterized by their abundance and community distribution alone (Hu et al., 2018). For example, the relative abundance of *Mortierellomycota* in this study was higher in reclaimed soils in different years. However, it accounted for a tiny proportion of the fungal network structure, indicating that the phylum had few significant connections with other species. In this study, the diversity of microbial communities and the stability of microbial networks were not synchronized (Figure 1 and Table 2). Although CK had the highest Shannon and Simpson index, its network stability (avgK) was not the highest. With the increase in reclamation ages, the stability of fungal communities in reclaimed soils surpassed that of CK. Combined with the composition of fungal communities at the phylum and genus levels in Figure 3, we suggest that some of the fungi that emerged during the reclamation process may have contributed to this result. Some low-abundance fungi could be keystone members of the soil microbial community and likely play more essential roles in maintaining ecological stability and aggregating fungal communities than some higher-abundance genera (Zhang et al., 2022). Berry and Widder (2014) suggested that modular hubs and connectors within or between modules may be keystone species that play an important role in the microbial community system (Han et al., 2004; Montoya et al., 2006), often considered as keystone taxa, as their role may influence the microbial composition and network structure (Herren and Mcmahon, 2017; Fan et al., 2018).

Reclaimed soils will result in greater community stability and thus provide greater resistance to disturbance (Scheffer et al., 2012). A shorter average geodesic distance (GD) means more efficient transfer of material, energy, and information between species (Zhou et al., 2011). Among the five fungal MENs constructed in this study, Plot 1 had the highest GD (4.979), indicating that its soil fungi were the most sensitive and responsive to the external environment and more susceptible to changes in community structure. However, this fungal network structure may lead to significant changes in the community of fungal species involved in nutrient cycling of soil carbon, nitrogen, and phosphorus, affecting plant growth. In contrast, Plots 4 and 3 soil fungi respond more slowly, and perturbations of environmental factors do not affect the entire bacterial ecological network in a short period, and the community structure is more stable (Jeanbille et al., 2016). Besides, Morrien et al. (2017) suggested that a tighter network could improve effective carbon utilization. In our study, nutrient exchange between soil fungal species may have been enhanced after longer years of reclamation. The CK network was relatively more negatively linked, suggesting that these fungal species may be competing for resources (Fuhrman, 2009). However, there was no intense competition in the reclaimed soils and even more heterogeneous ecological niches for soil fungi (Banerjee et al., 2016).

The presence of more interconnected members is beneficial to the fungal community. Like human societies, more interconnected individuals in the society mean that more events of cooperation and communication occur (Sun et al., 2016). As a result, society works more efficiently, and social goals are more likely to be achieved. In this case, an apparent effect can be observed in the form of more vigorous growth of restored vegetation (Figure 1). We can also understand the process of change in the fungal molecular ecological network with increasing years of reclamation by looking at the response of most fungal community members to soil variables. The majority (70%) of modules in Plot 4 soils was stimulated (encouraged) by certain soil variables, while the majority (56%) of Plot 1 soils was inhibited (discouraged) (Figure 6). For a complex natural community, every environmental factor cannot encourage every community member: most are meaningful (Mougi and Kondoh, 2012; Sun et al., 2016). From this point of view, a soil that has undergone longer years of reclamation is likely to be a soil with variables that encourage most fungal communities, whereas a soil at the beginning of reclamation is soil with variables that discourage most fungal communities. Any community that encourages the majority of its members is more likely to be a vibrant and successful community.

### Limitations and Prospects for Ecological Restoration of Mines in Eco-Fragile Regions

This study applied molecular ecological network to outline the internal network connection of each soil fungal community and its association network with soil physicochemical properties, explored the structural changes of soil fungal communities after coal mining reclamation and the response of their molecular ecological network, and proposed the laws in the process of fungal community succession. It provides a theoretical basis for revealing soil fungal community structure changes and mechanisms after mine reclamation. It provides a new idea for monitoring soil reconstitution and ecological restoration in mine areas: establishing a molecular ecological network monitoring system for soil fungi in mine reclamation.

Ecosystem monitoring in mining areas is an important tool to cope with environmental changes and has been a key construction project. We explored the succession of soil fungal communities under four different reclamation ages 14 years after reclamation and found some remarkable patterns. For example, the fungal abundance and diversity increased gradually with the increase in reclamation ages, and the correlation between fungal community structure changes and soil NN, C/N ratio, pH, and SWC also increased gradually with the change of ages. The fungal MENs also succeed with the natural vegetation recovery, with larger modules, more positive connections, tighter networks, and increased resistance to environmental changes. The formation of large modules with more than 60 nodes appeared in the ninth year after reclamation, and the results from Lefse analysis (Figure 3B) can be used by *GS14* (phylum to species) and *f_Nectriaceae* as marker genes. These obvious patterns can express the environmental microscopic changes of reclaimed soils quantitatively and visually at regular intervals and can be well monitored to assess the ecological succession patterns after the reclamation of mining areas. This helps rationalize the land reclamation and ecological restoration strategies and guide the soil reconstitution toward a reasonable evolutionary direction.

Since this study adopts the “space for time”approach, other potentially influencing factors such as special meteorological conditions, seasonal changes, and reclamation techniques are still unknown as a “black box” for up to 14 years. Although the spatial location of sampling points is not large, the results of this study need to be further deepened due to the spatial heterogeneity of soil. In addition, the differences in coal mining technology, the intensity of coal mining disturbance, and the seasonal classification of coal mining disturbance were not considered in this study and need to be further strengthened in the later stage.

## Conclusion

Our study demonstrates that the fungal community succession did not along the original but rather the direction of secondary succession in a coal mining subsidence after reclamation. By Illumina high-throughput sequencing, we found that reclamation significantly impacted soil fungi, with fungal abundance and community composition different from those of unmined soils. Reclamation could gradually increase fungal community abundance and diversity over time, but the dominant group unchanged. The main soil environmental factors affecting the distribution of fungal communities at different reclamation years were NN, EC, SWC, C/N ratio, TN, and pH. The succession of fungal MENs was clearly observed, and several indicators showed regularity over time, indicating that the analysis of fungi can be applied to the evaluation of ecological restoration projects in eco-fragile regions. In particular, most soil physicochemical properties facilitated the formation of large modules in the network. These findings should increase our understanding of soil fungal responses to coal mining reclamation and provide essential insights for developing scientifically sound reclamation and assessment plans.

## Data Availability Statement

The datasets presented in this study can be found in online repositories. The names of the repository/repositories and accession number(s) can be found below: www.ncbi.nlm. nih.gov/, PRJNA761520.

## Author Contributions

CJ completed the writing of the manuscript, data processing, data analysis, and summary of results. JH provided guidance and sublimation on the background, and significance of the study. HY contributed to the framework and logical structure of the manuscript. XR checked and revised the English grammar and spelling of the thesis. YT completed the implementation of the experiments and the collection of samples. XZ provided the experimental apparatus and gave guidance on the structure of the thesis. All authors contributed to the article and approved the submitted version.

## Conflict of Interest

The authors declare that the research was conducted in the absence of any commercial or financial relationships that could be construed as a potential conflict of interest.

## Publisher’s Note

All claims expressed in this article are solely those of the authors and do not necessarily represent those of their affiliated organizations, or those of the publisher, the editors and the reviewers. Any product that may be evaluated in this article, or claim that may be made by its manufacturer, is not guaranteed or endorsed by the publisher.

## Footnotes

1
http://data.cma.cn

2
http://ieg2.ou.edu/MENA
